# Open Access to Antipsychotics in State Medicaid Programs: Effect on Healthcare Resource Utilization and Costs among Patients with Serious Mental Illness

**DOI:** 10.36469/001c.137909

**Published:** 2025-06-17

**Authors:** Rashmi Patel, Onur Baser, Heidi C. Waters, Daniel Huang, Leigh Morrissey, Katarzyna Rodchenko, Gabriela Samayoa

**Affiliations:** 1 University of Cambridge; 2 Graduate School of Public Health City University of New York; 3 Department of Economics Boğaziçi University, Istanbul, Turkiye; 4 Otsuka Pharmaceutical Development & Commercialization, Inc., Princeton, New Jersey, USA; 5 Columbia Data Analytics, New York, New York, USA

**Keywords:** Medicaid, antipsychotics, serious mental illness, cost, utilization, open access

## Abstract

**Background:** The restrictive consequences of Medicaid formulary restriction policies on antipsychotic medications may lead to higher healthcare utilization and costs among beneficiaries with serious mental illness (SMI). **Objectives:** This study compared outcomes among patients with SMI accessing antipsychotic medications through state Medicaid programs with open access (OA) policies (Michigan) vs 5 states without Medicaid OA policies (California, Colorado, Florida, Illinois, Wisconsin). **Methods:** A retrospective analysis was conducted using Kythera Labs Medicaid data (Jan. 1, 2016–Dec. 31, 2023). Outcomes were assessed for patients with SMI (>18 years of age, ≥1 antipsychotic medication claim during the identification period (Jan. 1, 2017–Dec. 31, 2022), ≥1 SMI claim in the 12-month baseline). Continuous medical and pharmacy benefits were required for 12 months pre- and post-index date. Outcomes included SMI-related hospital admissions, length of hospital stay, emergency department and outpatient visits, and associated costs. **Results:** A greater proportion of beneficiaries with SMI resided in Michigan than in the other states. After matching, significantly more antipsychotics users experienced SMI-related hospitalizations in California (18.25% vs 9.47%, P < .0001), Colorado (11.41% vs 7.33%, P  =.0004), Florida (19.70% vs 10.17%, P < .0001), Illinois (23.57% vs 8.79%, P < .0001), and Wisconsin (15.21% vs 10.02%, P = .0046) than in Michigan. Length of stay was lower in Michigan than in California, Colorado, and Illinois. Inpatient costs related to SMI were significantly lower in Michigan, yet pharmacy costs were higher. Total SMI-related costs were higher in all non-OA states than in Michigan, except Colorado. **Discussion:** State Medicaid programs without OA to antipsychotics were associated with higher rates of SMI-related resource utilization and costs vs Michigan. **Conclusions:** Policy makers should consider the potential downstream cost implications of restrictive access policies and evaluate whether OA could result in improved health outcomes for patients and savings for Medicaid programs.

## INTRODUCTION

Medicaid, the federal-state health insurer for low-income individuals, now provides coverage for more than one-fifth of Americans with mental health disorders.^1^ Recently, growth in Medicaid prescription drug expenditures has outpaced trends observed in other healthcare services, emerging as one of the primary contributing factors to overall increases in program costs.^2^ Net spending on Medicaid prescription drugs increased by 47% between 2017 and 2022, from $29.8 billion to $43.8 billion.^3^ To address the challenges associated with funding prescription medications, some state Medicaid programs often implement prior authorization and step therapy as utilization management strategies.^4^ Prior authorization allows reimbursement for medications only if the prescriber requests and secures advance approval from Medicaid.^5^ Step therapy is a payer-developed regimen of “steps” that requires patients to use and demonstrate a lack of response to lower-cost medications before use of non-preferred, more costly medications is approved.^5^

Given rising healthcare costs and increasing pressure on budgets, “atypical” or second-generation APs and other psychotherapeutic drugs are increasingly subject to these restrictions. For example, since 2015, 31 state Medicaid programs implemented prior authorization for antipsychotic medications (APs) prescribed to Medicaid-enrolled youth, and 15 states incorporated clinical review and other quality monitoring programs.^6^

Colorado, Florida, California, Illinois, and Wisconsin have some of the most restrictive Medicaid pharmacy programs for patients with SMI, imposing utilization management strategies such as age limits, quantity caps, dose optimization, as well as prior authorization and/or step therapy. In contrast, Michigan’s Medicaid program does not impose restrictions and instead provides open access (OA) to psychiatric drugs.

The expanded implementation of these restrictions imposes challenges on both physicians and patients. A 2023 survey of 1000 practicing physicians across various specialties revealed that, on average, they completed 43 prior authorizations for medications and procedures per week, dedicating approximately 12 hours to this task. Another report indicated that 55% of patients reported delays in therapy due to a medication requiring prior authorization.^7^ The impact is pronounced for patients with SMI, who often require multiple medication adjustments involving polypharmacy to find the most effective treatment due to individual variability in response and side effects.^8,9^ This complex treatment process, combined with prior authorization requirements, may explain why the implementation of utilization management on prescription drugs indicated for conditions such as diabetes, depression, schizophrenia, and bipolar disorder has been correlated with a deterioration in disease status and an increase in hospitalization rates and overall net medical expenditures.^10,11^

Since half of Medicaid beneficiaries with SMI report unmet needs,^12^ the purpose of this study is to demonstrate to stakeholders the fiscal benefits of open utilization of APs among patients with SMI. This study compared differences in health costs and outcomes between the less restrictive (OA) Medicaid policies in Michigan and the more restrictive (non-OA) policies in states such as Colorado, Florida, California, Illinois, and Wisconsin. It is hypothesized that non-OA AP policies would not only be associated with worse clinical outcomes for individuals with SMI but would also increase the long-term costs to Medicaid plans.

## METHODS

### Data Sources

We employed a retrospective cohort design to analyze Kythera Medicaid files from January 2016 to December 2023. Kythera data encompass medical and pharmacy claims and represent coverage of 79% of the US patient population.^13^ The data set includes both open and closed versions, encompassing approximately 310 million patients, 6.1 million practitioners, 1.6 million organizations, and 1.4 million facilities that collectively generate 40 billion healthcare claims.^14^ Kythera contains commercial, Medicare, and Medicaid claims. We utilized the Medicaid closed portion, which contains data for 44 470 509 patients and 901 136 733 claims. The data set includes de-identified patient ages, genders, types of insurance (fee-for-service vs managed care), zip codes, diagnoses according to the *International Classification of Diseases*, *Tenth Revision* (ICD-10), Current Procedural Terminology codes, and National Drug Codes for medications. Each patient is assigned a unique identifier that links their encounters, allowing for longitudinal analysis. Details of the data have been published elsewhere, and the healthcare outcomes derived from these data have been compared with other data sets for validity and consistency.^15–17^

### Handling of Missing Data

Patients included in the Kythera databases are generally complete and rarely have missing data. In the event of missing information, patients are benchmarked based on their characteristics and enrollment status and cross-referenced with other data sources to ensure accuracy. Claims with missing elements, such as gender or birth year, are retained only if these omissions do not impact the study outcomes. Any remaining missing data that could affect the analysis are excluded from the study.

The raw data set on which this study is based is available through a commercial data licensing agreement with Kythera Labs. Kythera data have been expertly determined by Datavant’s Privacy Hub (Mirador) to comply with statistical de-identification required by the Health Insurance Portability and Accountability Act (HIPAA) and associated regulations. The analysis of de-identified, publicly available data does not constitute human subjects research as defined by US Department of Health and Human Services, 45 CFR § 46.102, and does not require Institutional Review Board (IRB) review.

### Study Design and Population

Retrospective claims were used to examine primary outcomes related to healthcare costs and utilization. During the follow-up period, both all-cause and SMI-specific healthcare costs were calculated, encompassing outpatient, inpatient, emergency department (ED), and pharmacy expenses. These costs were adjusted to 2023 US dollars using the medical care component of the Consumer Price Index. Healthcare utilization for both all-cause and SMI-specific cases was assessed by measuring the number of inpatient admissions, ED visits, outpatient visits, and length of hospital stay (LOS).

The study period was from January 1, 2016, through December 31, 2023. The date of the first prescription claims for AP treatment (index date) during the identification period, from January 1, 2017, through December 31, 2022, was considered the first-line treatment initiation. Continuous health plan enrollment for 12 months pre- and 12 months post-index was required. Eligible patients were required to be at least 18 years of age.

The population with SMI in each of the selected states was defined by a diagnosis of schizophrenia or its related spectrum disorders, including schizoaffective disorder, schizophreniform disorder, bipolar disorder, mania and associated disorders, major depressive disorder with recurrent episodes, and specific personality disorders 1 year pre-index date. Patients were excluded if they were prescribed any AP during the baseline period, were prescribed clozapine during the study period, or were dually eligible for Medicaid and Medicare.

### Analysis

The study utilized propensity score matching (PSM) to compare healthcare utilization and outcomes, consisting of the Michigan Medicaid program with a mental health OA policy, and the case cohort, comprising the non-OA Colorado, Florida, California, Illinois, and Wisconsin Medicaid programs. Propensity score matching is widely employed in observational studies for causal inference.^18^ The propensity score represents the estimated probability of being a member of the case cohort, given a specific covariate pattern.^19^ Each subject in the case cohort was matched one-to-one with a subject in the control cohort with the closest propensity score. The matching process utilized patient demographics, claims-based measures of patient health, and medication characteristics at baseline as covariates.

The Kythera Medicaid data provided fundamental demographic information, including age and gender. The status of the index AP as preferred or non-preferred was determined based on each state’s Medicaid formulary. Given the variability in Medicaid formularies across states, APs listed as preferred agents on the preferred drug lists in the non-OA states were categorized as preferred, while those listed as non-preferred were categorized accordingly for each state. To facilitate comparison between AP users in Michigan and those in non-OA states, Michigan was assigned the same sets of APs as the comparison state. The preferred and non-preferred APs included in the states’ preferred drug lists are detailed in **Supplemental Tables S1-S5**.

To account for variations in comorbidities among patients, we utilized 3 comorbidity indices: the revised Charlson Comorbidity Index (CCI), the Chronic Disease Score (CDS), and the Elixhauser Index. The CCI, developed to predict long-term mortality, has been adapted for use with various data sources, including ICD-9 and ICD-10 codes, and is known for its reliability and validity in diverse clinical populations.^20^ The CDS, a drug-based index, stratifies patients based on prescription data to predict health outcomes.^21^ The Elixhauser Index categorizes comorbidities using ICD codes and is widely used for risk adjustment in health services research.^22^ The application of these indices within a matching algorithm has been demonstrated to enhance the robustness of estimators. We also controlled for mental and systemic comorbidities.

Covariates were subjected to descriptive analysis. For categorical variables, frequencies and percentages were reported, while for continuous variables, means and SD were calculated. To determine statistically significant differences between the cohorts, *t*-tests were applied to continuous variables, and Pearson’s $\chi^{2}$ tests were used for categorical variables, with a significance threshold set at the 5% level. Standardized differences were computed for each variable. Post-PSM, it is anticipated that there will be no significant differences in all pre-index measures between the 2 patient cohorts. All analyses were conducted using Pyspark and SparkR on Databricks and R.

## RESULTS

After applying inclusion and exclusion criteria, the analysis included 7129 patients with SMI using APs from California, 4233 from Colorado, 2369 from Florida, 3558 from Illinois, 1374 from Wisconsin, and 4066 from Michigan (**Supplemental Figure S1**).

The percentage of Medicaid beneficiaries with SMI varied. Michigan had a higher percentage of Medicaid beneficiaries with SMI than other states, and the percentage of Medicaid beneficiaries with SMI increased from 2016 to 2023 in all states except Florida (**Supplemental Figure S2**).

Michigan had the youngest patient cohort, and California and Florida had the oldest. Michigan also exhibited the highest proportion of female beneficiaries with SMI, followed by Wisconsin. California recorded the highest CCI score, indicating a more significant burden of physical comorbidities. Michigan had the highest CDS, suggesting an elevated medication burden. Furthermore, California had the highest Elixhauser Index score, reflecting more comorbidities. California’s beneficiaries were found to have more physical comorbidities, while Wisconsin exhibited higher rates of specific mental health comorbidities. Additionally, Michigan and California had the highest rates of non-preferred AP use among patients with SMI, surpassing the rates observed in Illinois, Wisconsin, Colorado, and Florida (**Table 1**).

**Table 1.** Baseline Demographic and Clinical Characteristics

**Characteristics**	**California** **(N = 7129)**	**Colorado** **(N = 3749)**	**Florida** **(N = 2036)**	**Illinois** **(N = 2851)**	**Wisconsin** **(N = 1316)**	**Michigan** **(N = 4066)**
Mean age (SD)	42.31 (14.8)	39.35 (12.9)	42.23 (14.5)	41.88 (13.9)	40.03 (12.9)	39.01 (13.0)
Female (%)	3926 (55.1)	2209 (58.9)	1142 (56.1)	1585 (55.6)	802 (60.9)	2571 (63.2)
Non-preferred AP use (%)	1810 (25.4)	424 (11.3)	166 (8.2)	592 (20.8)	159 (12.1)	1062 (26.1)
Comorbidity scores (SD)						
CCI score	1.04 (1.5)	0.48 (0.9)	0.83 (1.3)	0.89 (1.3)	0.77 (1.2)	0.83 (1.3)
CDS	2.35 (2.8)	2.49 (2.7)	2.39 (2.9)	2.67 (2.9)	2.94 (3.1)	3.58 (3.1)
Elixhauser Index score	4.02 (3.2)	2.60 (2.1)	3.71 (2.9)	3.74 (2.9)	3.57 (2.88	3.43 (2.8)
Comorbidities						
Mental health comorbidities						
Depression	131 (29.9)	794 (21.2)	629 (30.9)	983 (34.5)	494 (37.5)	1559 (38.3)
Substance use disorder	2407 (33.8)	1329 (35.5)	656 (32.2)	1046 (36.7)	532 (40.4)	1332 (32.8)
Substance-induced psychotic behavior	95 (1.3)	43 (1.2)	29 (1.4)	28 (1.0)	14 (1.1)	18 (0.4)
Specific personality disorder	137 (1.9)	97 (2.6)	69 (3.4)	75 (2.6)	69 (5.2)	103 (2.5)
Borderline personality disorder	299 (4.2)	204 (5.4)	74 (3.6)	217 (7.6)	116 (8.8)	234 (5.8)
Post-traumatic stress disorder	961 (13.5)	907 (24.2)	246 (12.1)	527 (18.5)	370 (28.1)	645 (15.9)
Severe agitation	67 (0.9)	27 (0.7)	12 (0.6)	22 (0.8)	16 (1.2)	76 (1.9)
Dementia	185 (2.6)	33 (0.9)	46 (2.3)	52 (1.8)	18 (1.4)	57 (1.4)
Delirium	201 (2.8)	52 (1.4)	38 (1.9)	75 (2.6)	24 (1.8)	79 (1.9)
Systemic comorbidities						
Type 2 diabetes	1255 (17.6)	278 (7.4)	320 (15.7)	473 (16.6)	180 (13.7)	527 (13.0)
Other specified diabetes mellitus	53 (0.7)	7 (0.2)	18 (0.9)	34 (1.2)	15 (1.1)	34 (0.8)
Viral hepatitis	387 (5.4)	92 (2.5)	118 (5.8)	124 (4.4)	46 (3.5)	176 (4.3)
Constipation	629 (8.8)	191 (5.1)	175 (8.6)	195 (6.8)	81 (6.2)	345 (8.5)
Parkinson’s disease	51 (0.7)	5 (0.13)	12 (0.6)	8 (0.3)	3 (0.2)	12 (0.3)
Chronic obstructive pulmonary disease	714 (10.0)	209 (5.6)	241 (11.8)	315 (11.1)	114 (8.7)	416 (10.2)
Chronic pain syndrome	413 (5.8)	149 (4.0)	114 (5.6)	87 (3.1)	69 (5.2)	217 (5.3)
Atrial fibrillation	185 (2.6)	29 (0.8)	50 (2.5)	58 (2.0)	16 (1.2)	75 (1.8)
Hypertension	2414 (33.9)	617 (16.5)	766 (37.6)	1014 (35.6)	373 (28.3)	1284 (31.6)
Coronary heart disease	387 (5.4)	58 (1.6)	133 (6.5)	182 (6.4)	54 (4.1)	185 (4.6)
Peripheral vascular disease	120 (1.7)	22 (0.6)	44 (2.2)	45 (1.6)	19 (1.4)	82 (2.0)

Abbreviations: AP, antipsychotic; CCI, Charlson Comorbidity Index; CDS, Chronic Disease Score.

Following PSM, the number of patients with SMI using APs matched with Michigan was 3726 in California, 2550 in Colorado, 1838 in Florida, 2423 in Illinois, and 1308 in Wisconsin.

**Figure 1.**
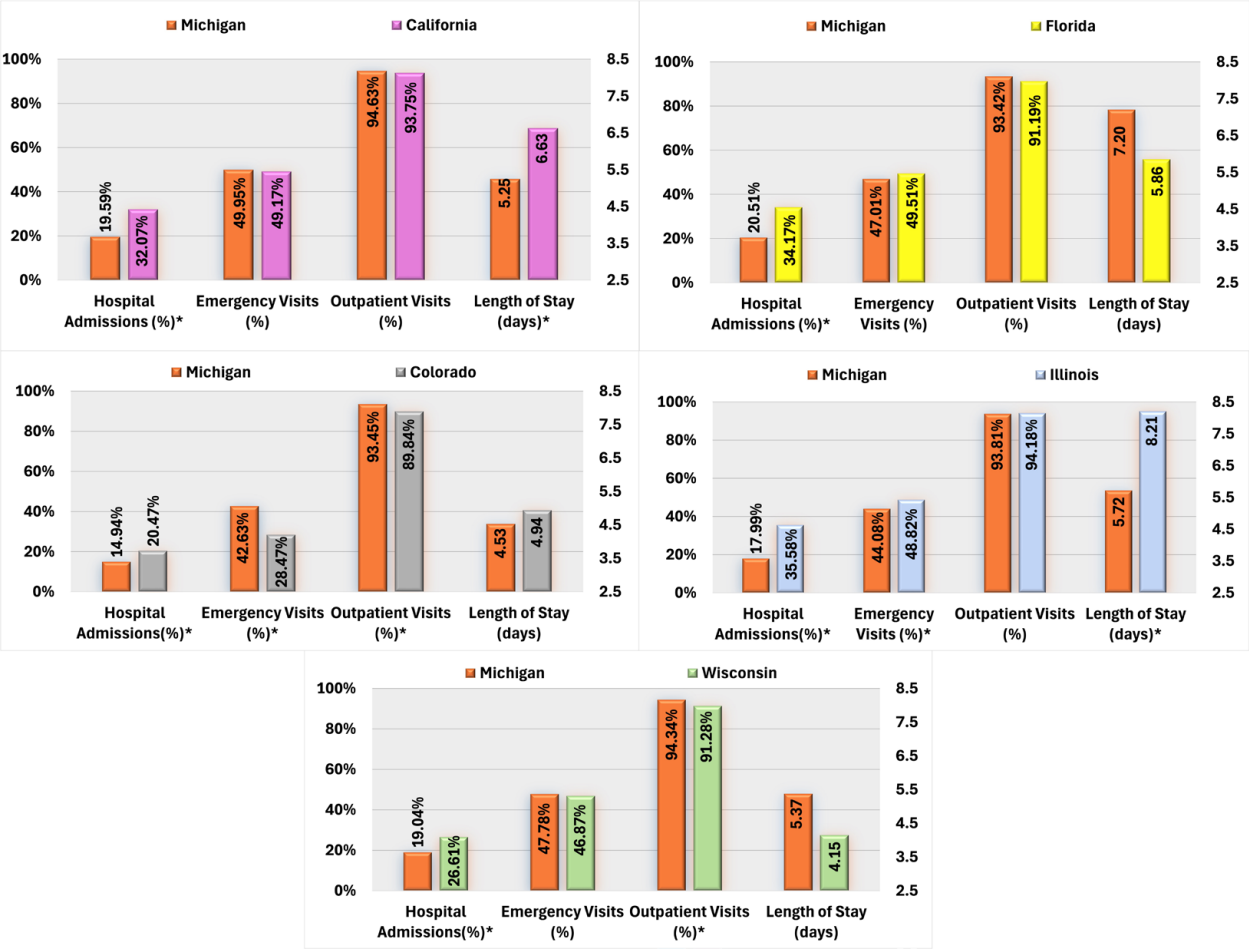
PSM-Adjusted Comparison of All-Cause HCRU Among Medicaid Beneficiaries With SMI Using AP in Michigan vs California, Colorado, Florida, Illinois, and Wisconsin Abbreviations: AP, antipsychotic; HCRU, healthcare resource utilization; SMI serious mental illness. *Significant at *P* < .05.

Regarding all-cause healthcare resource utilization, patients with AP use in Michigan demonstrated significantly lower hospital admission rates and shorter average hospital stays than those in California. Conversely, AP users in Colorado exhibited higher rates of hospital admissions but lower proportions of ED and outpatient visits than those in Michigan. Furthermore, Michigan AP users were less likely to be hospitalized than Florida users, and compared with Illinois, Michigan AP users had lower rates of ED visits and hospital admissions, and shorter average LOS. Finally, Michigan AP users exhibited a lower overall hospital admission but a higher proportion of outpatient visits than those in Wisconsin (**Figure 1**).

**Figure 2.**
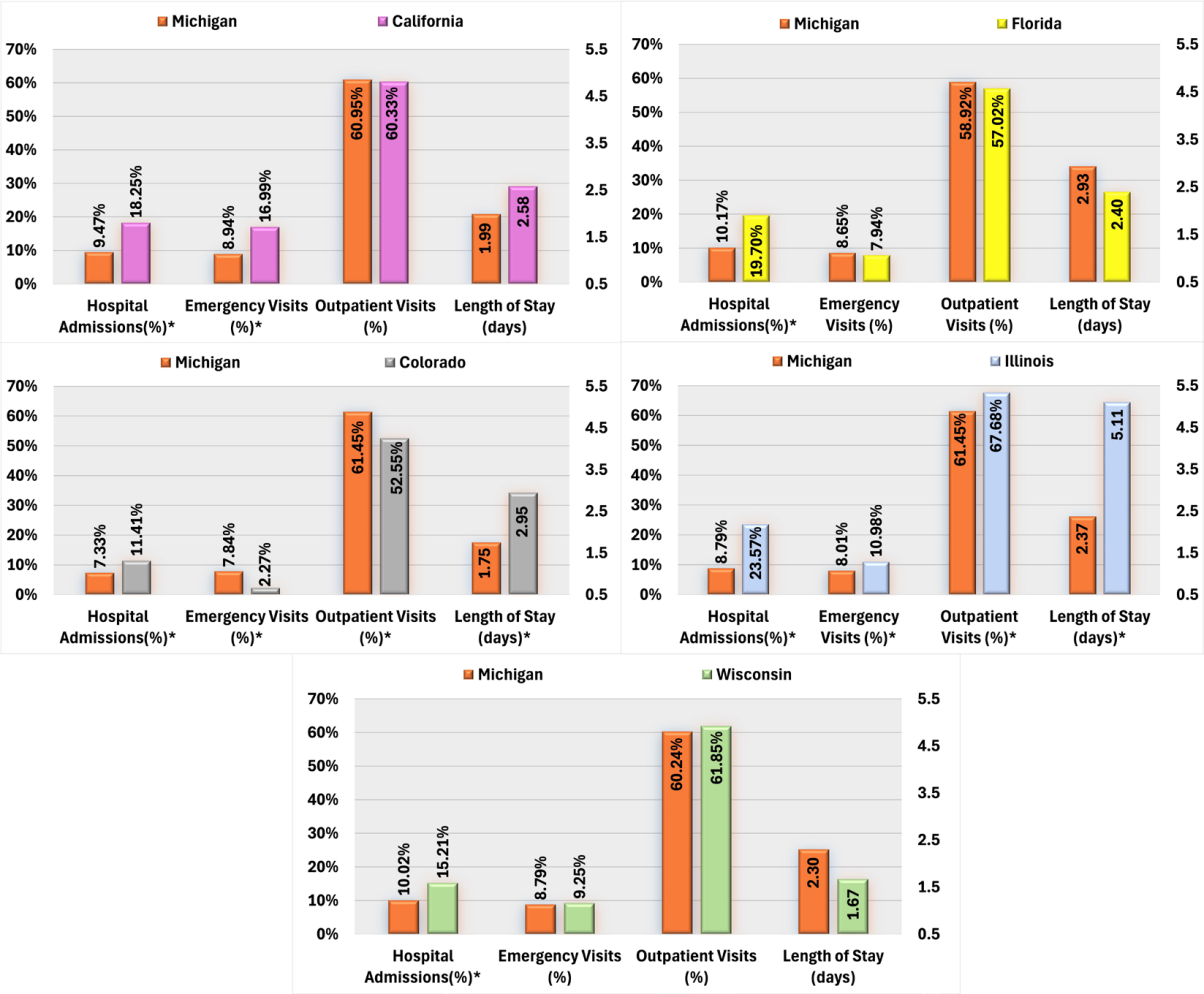
PSM-Adjusted Comparison of SMI-Related HCRU Among Medicaid Beneficiaries Using AP in Michigan vs California, Colorado, Florida, Illinois, and Wisconsin Abbreviations: AP, antipsychotic; SMI, serious mental illness. *Significant at *P* < .05.

Compared with California, Michigan AP users were significantly less likely to experience SMI-related hospital admissions and ED visits. Conversely, AP users in Colorado were more likely to be hospitalized for SMI-related reasons and had longer hospital stays but exhibited a lower proportion of ED visits and outpatient visits than the Michigan cohort. Michigan AP users also had lower rates of SMI-related hospital admissions than those in Florida and Wisconsin. Additionally, Michigan AP users demonstrated significantly lower SMI-related HCRU than those in Illinois, including hospital admissions, LOS, ED, and outpatient visits (**Figure 2**).

State Medicaid programs without OA to APs were associated with higher rates of SMI-related resource use and cost than Michigan, including a higher proportion of hospital admissions (**Supplemental Figure S3**).

**Figure 3.**
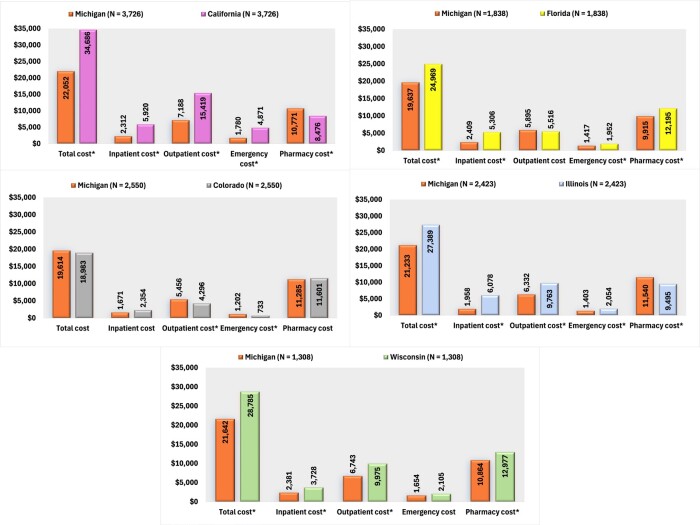
PSM-Adjusted All-Cause Costs Among Medicaid Beneficiaries With SMI Using AP in Michigan vs California, Colorado, Florida, Illinois, and Wisconsin Abbreviations: AP, antipsychotic; SMI, serious mental illness. *Significant at *P* < .05.

In an analysis of all-cause healthcare costs, Michigan demonstrated significantly lower expenditures than California across several categories: inpatient costs, outpatient costs, and ED costs. Despite Michigan incurring slightly higher pharmacy costs, the overall healthcare costs remained significantly lower. Compared with Colorado, there were no significant differences in total, inpatient, and pharmacy costs, although Michigan’s outpatient and ED costs were higher. Patients residing in Michigan also incurred lower inpatient and total costs than Florida, along with reduced ED and pharmacy costs. Compared with Illinois, Michigan’s inpatient, outpatient, and ED costs were lower, although pharmacy costs were slightly elevated. Nevertheless, Michigan’s overall costs were significantly lower. Lastly, Michigan’s healthcare costs were lower than Wisconsin’s in inpatient, outpatient, pharmacy, and total costs (**Figure 3**).

**Figure 4.**
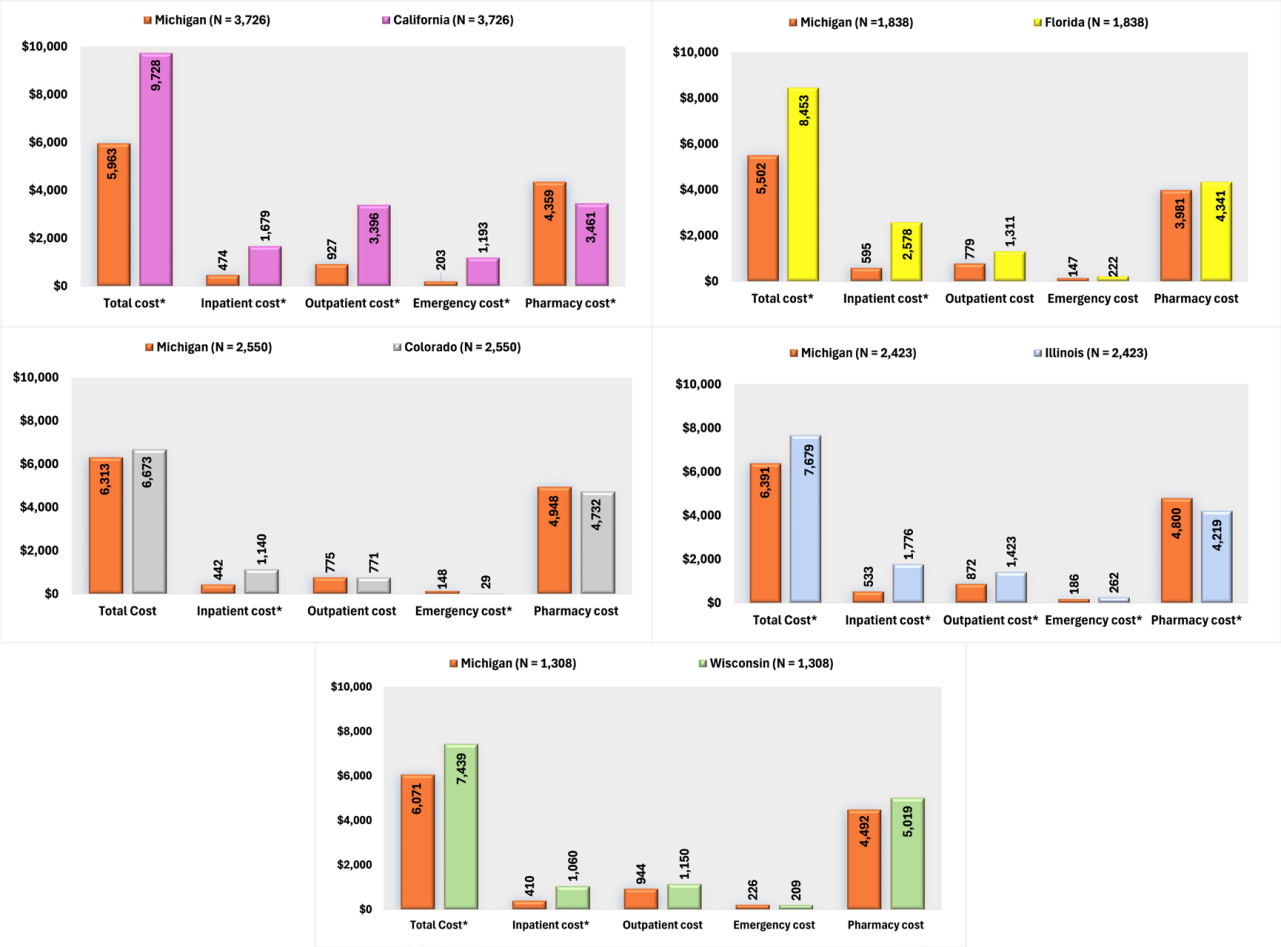
PSM-Adjusted SMI-Related Costs Among Medicaid Beneficiaries Using AP in Michigan vs California, Colorado, Florida, Illinois, and Wisconsin Abbreviations: AP, antipsychotic; SMI, serious mental illness. *Significant at *P* < .05.

In terms of SMI-related costs, Michigan patients incurred significantly lower SMI-related expenditures than those in California, with reduced costs across inpatient, ED, and outpatient services, resulting in notably lower total costs. Michigan patients also incurred reduced inpatient and total costs compared with Florida. Compared with Illinois, Michigan had lower inpatient, ED, and outpatient costs, although pharmacy costs were slightly higher. Total SMI-related costs remained lower in Michigan than in Illinois. Furthermore, inpatient and total costs were lower in Michigan than in Wisconsin (**Figure 4**).

## DISCUSSION

This study assessed outcomes among patients with SMI who were using APs within a state Medicaid program that implements OA policies for APs (Michigan) against those in states without such policies (California, Colorado, Florida, Illinois, and Wisconsin). We found that patients prescribed atypical AP medications experienced significantly poorer outcomes in states without OA policies. Specifically, these patients faced an increased risk of both overall and SMI-related hospitalizations, as well as higher overall and SMI-related medical costs.

These findings are consistent with an expanding body of evidence indicating that formulary restrictions can lead to unintended consequences, including burdens on provider and patient time, increased rates of treatment discontinuation, and reduced overall quality of care. For instance, one study reported a 50% increase in the risk of mental health hospitalization following a missed prescription refill for individuals with schizophrenia.^23^

This study found that, following the implementation of formulary restrictions, California and Illinois experienced modest cost savings, while Colorado demonstrated no significant difference in pharmacy spending. These results align with previous research, which has consistently shown that formulary restrictions for APs lead to minimal reductions in pharmacy expenditures^24,25^ or have no discernible impact on overall pharmacy costs,^14,26^ yet also lead to more significant increases in HCRU and treatment discontinuation.^27^ Our study showed that, even in states that experienced modest savings in pharmacy costs, these savings were outweighed by increased expenditures in inpatient, outpatient, and emergency care services.

Interestingly, the Colorado cohort had a significantly higher proportion of SMI-related inpatient costs, but not total SMI or all-cause costs compared with Michigan. While Colorado does not have OA policies for oral APs, all long-acting injectable APs are on the state’s preferred drug list,^28^ which may have led to some of the similarities between Colorado and Michigan. Administrative costs and the potential loss of additional rebates from manufacturers for branded medications due to non-OA formulary policies may further exacerbate the financial burden on states. From a global perspective, individual US states allocate a significantly larger portion of their health expenditures to administrative tasks than nearly any other country.^29^

Given the observational nature of this study, causality cannot be definitively established. Alternative explanations for cost and utilization differences may include variations in mental health provider availability, state-level mental health infrastructure, or other Medicaid-related policy differences not directly accounted for, such as differences in provider reimbursement rates.

While open access to APs can reduce barriers to medication access and potentially improve outcomes, it is also important to consider potential unintended consequences. For instance, increased medication access might inadvertently lead to higher rates of off-label prescribing or polypharmacy, a common concern within Medicaid populations.^30,31^ Future research should investigate the prevalence and impact of these phenomena in states with OA policies.

Several factors could explain the relationship between worse patient outcomes and formulary restrictions. First, the substantial barriers imposed by these restrictions can hinder patients from acquiring their initially prescribed medications. The lack of transparency in frequently changing formularies and prior authorization requirements often leaves prescribing physicians uncertain about which treatment options will be filled without delays. When patients attempt to retrieve their prescribed medications at pharmacies, they may unexpectedly find that further steps must be taken by their physicians to secure health plan approval. This marks the beginning of an intricate process that entails interactions with health plans, initial denials, formal written appeals, and “peer-to-peer” discussions with adjudicators who may lack familiarity with the specific disease or the medication in question, and who may propose unsuitable alternatives.^32^ According to one report, during this complex process of navigating formulary restrictions and prior authorization requirements, a staggering 37% of prescriptions that are initially rejected at the pharmacy are ultimately abandoned, never to be collected by the patients who need them.^33^ Second, atypical APs are a well-differentiated class of medications with varying mechanisms of action and side effect profiles.^34^ This variation necessitates personalized treatment plans to optimize efficacy and minimize adverse effects. Formulary restrictions that limit access to specific atypical APs can disrupt these personalized treatment plans. When patients are unable to access the most appropriate medication, they may experience suboptimal therapeutic outcomes, leading to increased rates of nonadherence and treatment discontinuation.

### Limitations

This study has several limitations that should be considered when interpreting the results. First, potential administrative costs and rebates associated with implementing and managing formulary restrictions were not accounted for. These costs could offset some of the savings from reduced pharmaceutical expenditures in California and Illinois and should be considered in a comprehensive evaluation of the policy’s impact. Second, the study considered the impact of formulary restrictions only on users of atypical APs. This focus excluded the potential broader effects of such restrictions on patients who might be deterred from initiating atypical AP therapy due to the restrictions. As a result, the study may underestimate the overall negative consequences of formulary restrictions. Third, the data used in this retrospective analysis were not nationally representative, which may limit the generalizability of the findings to other states or populations not included in the sample. The study focused on Medicaid beneficiaries from six states, and outcomes could potentially differ in states that were not part of the analysis. Fourth, the exclusion of dually eligible patients (eligible for both Medicare and Medicaid) could further limit the generalizability of the results. Dual-eligibility patients often have different healthcare needs and utilization patterns than those solely on Medicaid, which may influence the impact of formulary restrictions on healthcare outcomes and costs. Lastly, the analysis relied on claims data, which inherently have limitations such as potential inaccuracies in coding and the inability to capture all relevant clinical details. Claims data do not provide information on medication adherence or whether patients took the prescribed medications as directed, which could affect the observed outcomes.

Despite limitations, our claims data, which reflect the actual healthcare environment and capture real-world patient experiences and practices, encompass a vast cohort of patients using recent Medicaid data. This allowed us to effectively demonstrate the impact of formulary restrictions within the rapidly evolving landscape of atypical APs.

## CONCLUSION

State Medicaid programs that do not implement OA policies for APs are associated with higher rates of SMI-related HCRU and costs than Michigan, which has such policies. Policymakers should consider whether combining OA policies with additional measures, such as care coordination programs or adherence monitoring interventions, might maximize benefits while mitigating risks. Future policy development should address not only the financial aspects but also the holistic management of patients with SMI to improve both economic and health outcomes.

### Disclosures

H.C.W., D.H., and L.M. are employees of Otsuka; R.P. is a consultant to Otsuka. O.B. has no conflict of interest. K.R. is an employee of Columbia Data Analytics, which is a paid consultant to Otsuka. G.S. was an employee of Columbia Data Analytics at the time of the study.

## Supplementary Material

Online Supplementary Material
