# Supplementary material for: Open Access to Antipsychotics in State Medicaid Programs: Effect on Healthcare Resource Utilization and Costs among Patients with Serious Mental Illness
Source: J Health Econ Outcomes Res. 2025 Jun 17;12(1):222–9. doi: 10.36469/001c.137909 (PMC12178157; doi:10.36469/001c.137909)
Supplement: Online Supplementary Material [file jheor_2025_12_1_137909_289645.pdf]

Journal of Health Economics and Outcomes Research

Online Supplementary Material

Open Access to Antipsychotics in State Medicaid Programs: Effect on Healthcare Resource Utilization and Costs Among Patients With Serious Mental Illness. *JHEOR*. 2025;12(1):222-229. doi:10.36469/jheor.2025.137909

Figure S1: Attrition of Patients With Serious Mental Illness Using Antipsychotic Medication in California, Colorado, Florida, Illinois, Wisconsin, and Michigan

Table S1: California Preferred Drug List

Table S2: Colorado Preferred Drug List

Table S3: Florida Preferred Drug List

Table S4: Illinois Preferred Drug List

Table S5: Wisconsin Preferred Drug List

Figure S2: Proportion of Patients with Serious Mental Illness in the US Medicaid Population

Figure S3: PSM-Adjusted SMI-Related Hospital Admissions Among Medicaid Beneficiaries With SMI Using Antipsychotic Medications in Michigan, California, Colorado, Florida, Illinois, and Wisconsin

This supplementary material has been provided by the authors to give readers additional information about their work.

This is an open-access article distributed under the terms of the Creative Commons Attribution 4.0 International License (CCBY-4.0). View this license's legal deed at http://creativecommons.org/licenses/by/4.0 and legal code at http://creativecommons.org/licenses/by/4.0/legalcode for more information.

Figure S1. Attrition of Patients with SMI with AP Medication Use in California, Colorado, Florida, Illinois, Wisconsin, and Michigan

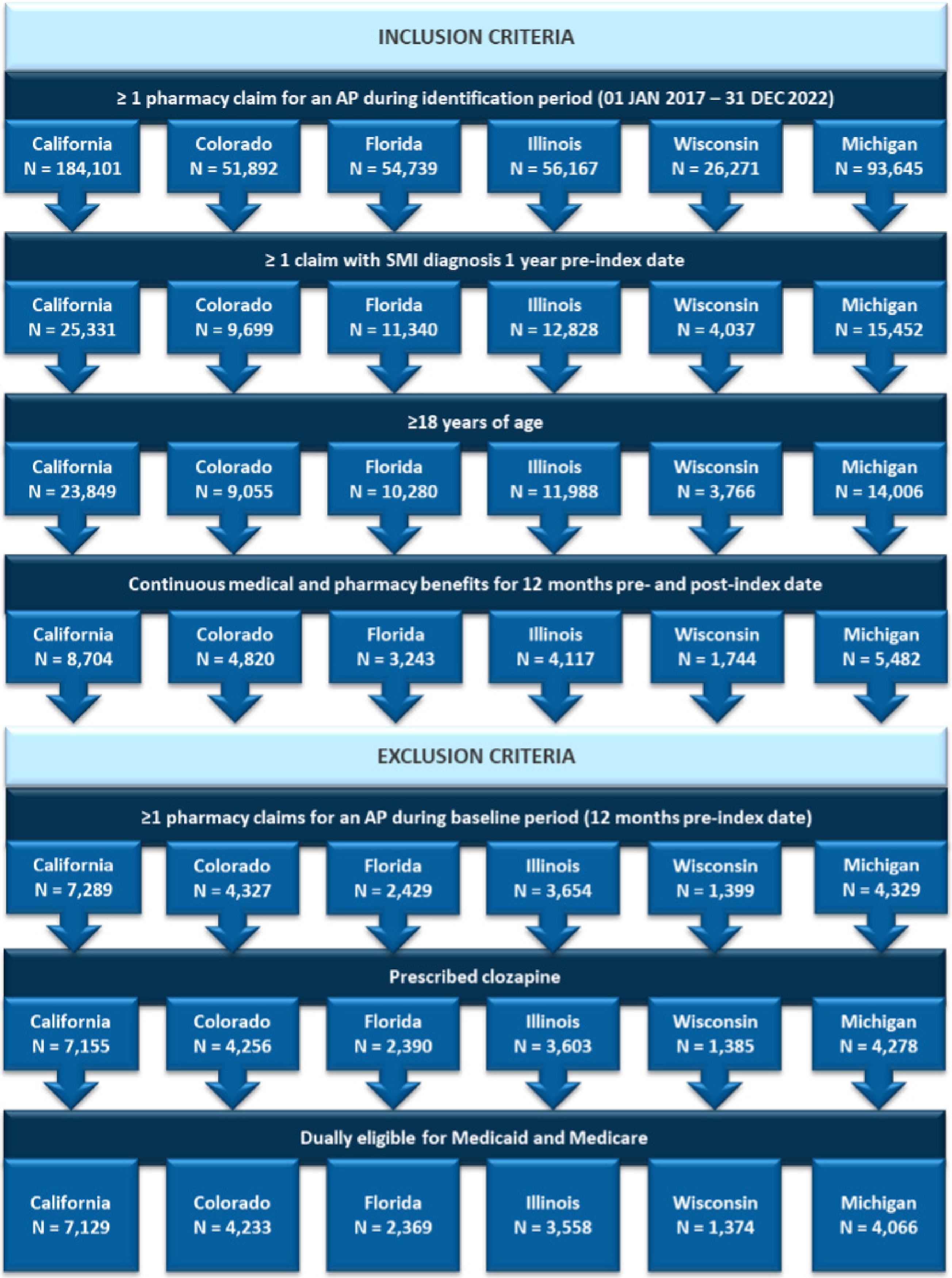

Abbreviations: AP, antipsychotic; SMI, serious mental illness.

Table S1. Attrition of Patients With Serious Mental Illness Using AP Medication in California, Colorado, Florida, Illinois, Wisconsin, and Michigan[8]

Non-preferred APs	Preferred APs
Abilify Asimtufii	Abilify tablet
Abilify Maintena	aripiprazole tablet, ODT, solution
Abilify MyCite	chlorpromazine tablets, oral concentrate
Adasuve	clozapine tablet, ODT
Aristada	Clozaril
Aristada Initio	Fanapt
asenapine	Fazaclo
Caplyta	fluphenazine HCL elixir, oral concentrate, tablet
chlorpromazine hydrochloride injection	Geodon capsule
fluphenazine decanoate	haloperidol liquid, tablets
fluphenazine hydrochloride injection	Invega tablet
Geodon injection	Latuda tablet
Haldol Decanoate	loxapine HCL solution
haloperidol decanoate	loxapine succinate capsule
haloperidol injection	lurasidone hydrochloride tablet
haloperidol lactate	molindone hydrochloride liquid, tablet
Invega Hafyera	olanzapine tablets, ODT
Invega Sustenna	paliperidone tablets, ER
Invega Trinza	perphenazine Injection, liquid, tablet
Lybalvi	quetiapine fumarate tablet, ER
Nuplazid	Risperdal solution, tablet
olanzapine and fluoxetine	risperidone solution, tablet
olanzapine injection	Saphris
Perseris	Seroquel
Pimozide	thioridazine liquid concentrate, tablet
Rexulti	thiothixene capsule, liquid
Risperdal consta	trifluoperazine tablet
risperidone vial, ODT	ziprasidone HCL capsule
Rykindo	Zyprexa tablet
Secuado	
Symbyax	
Uzedy	
Versacloz	
Vraylar	
ziprasidone mesylate vial	
Zyprexa Relprevv	
Zyprexa vial	
Zyprexa Zydis vial	

Abbreviations: AP, antipsychotic; ER, extended release; ODT, orally disintegrating tablet.
All lowercase letters = generic product; leading capital letter = brand name product.
Non-preferred agents require utilization management.

Table S2. Colorado Preferred Drug List[6]

Non-preferred APs	Preferred APs
Abilify (aripiprazole) tablet, MyCite	Abilify Maintena (aripiprazole)
aripiprazole oral solution, ODT	aripiprazole tablet
asenapine solution, tablet	Aristada (aripiprazole lauroxil)
Caplyta (lumateperone) capsule	Aristada Initio (aripiprazole lauroxil)
clozapine ODT	clozapine tablet
Clozaril (clozapine) tablet, ODT	Geodon (ziprasidone)
Fanapt (iloperidone) tablet, pack	Invega Hafyera (paliperidone palmitate)
Geodon (ziprasidone) capsule	Invega Sustenna (paliperidone palmitate)
Invega ER (paliperidone) tablet	Invega Trinza (paliperidone palmitate)
Latuda (lurasidone) tablet	lurasidone tablet
Lybalvi (olanzapine/samidorphan) tablet	olanzapine tablet, ODT
Nuplazid (pimavanserin) capsule, tablet	paliperidone ER tablet
olanzapine/fluoxetine capsule	Perseris (risperidone)
Rexulti (brexpiprazole) tablet	quetiapine ER tablet
Risperdal (risperidone) tablet, oral solution	quetiapine tablet
Secuado (asenapine) patch	Risperdal Consta (risperidone)
Seroquel IR (quetiapine IR) tablet	risperidone tablet, ODT, oral solution
Seroquel XR (quetiapine ER) tablet	Saphris (asenapine) solution, tablet
Symbyax (olanzapine/fluoxetine) capsule	ziprasidone capsule
Versacloz (clozapine) suspension	Zyprexa Relprevv (olanzapine pamoate)
Vraylar (cariprazine) capsule	
Zyprexa (olanzapine) tablet	
Zyprexa Zydis (olanzapine) ODT	

Abbreviations: AP, antipsychotic; ER, extended release; ODT, orally disintegrating tablet.
All lowercase letters = generic product; leading capital letter = brand name product.
Non-preferred agents require utilization management.

Table S3. Florida Preferred Drug List[12]

Non-preferred APs	Preferred APs
Abilify Asimtufii	aripiprazole solution, tablet
Abilify Maintena	chlorpromazine tablet, concentrate, injection
Aristada	Fanapt
Aristada Initio ER	fluphenazine decanoate
Caplyta	fluphenazine elixir, tablet, concentrate
clozapine tablet	haloperidol decanoate
Invega Hafyera	haloperidol lactate
Invega Sustenna	haloperidol tablet
Invega Trinza	loxapine capsule
Perseris	lurasidone tablet
Risperdal Consta	olanzapine tablet, ODT
Uzedy	perphenazine tablet
Vraylar	pimozide tablet
	quetiapine tablet
	risperidone tablet, ODT, solution
	thioridazine tablet
	thiothixene capsule
	trifluoperazine tablet
	ziprasidone capsule

Abbreviations: AP, antipsychotic; ER, extended release; ODT, orally disintegrating tablet.
All lowercase letters = generic product; leading capital letter = brand name product.
Non-preferred agents require utilization management.

Table S4. Illinois Preferred Drug List[7]

Non-preferred APs	Preferred APs
Abilify	aripiprazole tablet
Abilify Asimtufii	chlorpromazine tablet
Abilify Maintena	chlorpromazine concentrate
Abilify MyCite	fluphenazine elixir, tablet, concentrate
aripiprazole oral solution, ODT	clozapine tablet
Aristada	haloperidol concentrate, tablet
Aristada Initio ER	loxapine succinate capsule
asenapine solution	loxapine capsule
Caplyta	lurasidone hydrochloride tablet
clozapine ODT	olanzapine tablet, ODT
Clozaril	perphenazine tablet
Fanapt	quetiapine fumarate ER, tablet
Geodon	risperidone tablet, solution
Invega	thioridazine tablet
Invega Hafyera	thiothixene capsule
Invega Sustenna	trifluoperazine tablet
Invega Trinza	ziprasidone capsule
Latuda	
molindone hydrochloride	
Nuplazid	
olanzapine solution	
paliperidone ER	
Perseris	
Rexulti	
Risperdal Consta	
Risperdal solution, tablet	
risperidone ODT	
Saphris	
Secuado	
Seroquel	
Seroquel XR	
Uzedy	
Versacloz	
Vraylar	
ziprasidone mesylate vial	
Zyprexa Relprevv	
Zyprexa tablets, solution	
Zyprexa Zydis	

Abbreviations: AP, antipsychotic; ER, extended release; ODT, orally disintegrating tablet.
All lowercase letters = generic product; leading capital letter = brand name product.
Non-preferred agents require utilization management.

Table S5. Wisconsin Preferred Drug List[5]

Non-preferred APs	Preferred APs
Abilify Asimtufii	Abilify Maintena
Abilify MyCite	amitriptyline/perphenazine
Adasuve	aripiprazole ODT
asenapine (Gen-Saphris)	aripiprazole
Caplyta	Aristada Initio ER
clozapine ODT	Aristada
Fanapt	chlorpromazine
Fazaclo	clozapine
Latuda	fluphenazine decanoate
Lybalvi	fluphenazine
molindone tablet	Haldol Decanoate
Nuplazid	haloperidol decanoate
olanzapine/fluoxetine	haloperidol
paliperidone ER tablet	Invega Hafyera
quetiapine 150mg	Invega Sustenna
Rexulti	Invega Trinza
Rykindo ER	loxapine
Saphris	lurasidone (Gen-Latuda)
Secuado patch	olanzapine ODT, tablet
Symbyax	perphenazine
thioridazine	Perseris ER
Uzedy ER	pimozide
Versacloz	quetiapine fumarate ER
ziprasidone vial	quetiapine 25mg, 50mg, 100mg,200mg,300mg,400mg
	Risperdal Consta
	risperidone
	thiothixene
	trifluoperazine
	Vraylar
	ziprasidone capsules
	Zyprexa Relprevv

Abbreviations: AP, antipsychotic; ER, extended release; ODT, orally disintegrating tablet.
All lowercase letters = generic product; leading capital letter = brand name product.
Non-preferred agents require utilization management.

Figure S2. Proportion of Patients with SMI in the US Medicaid Population

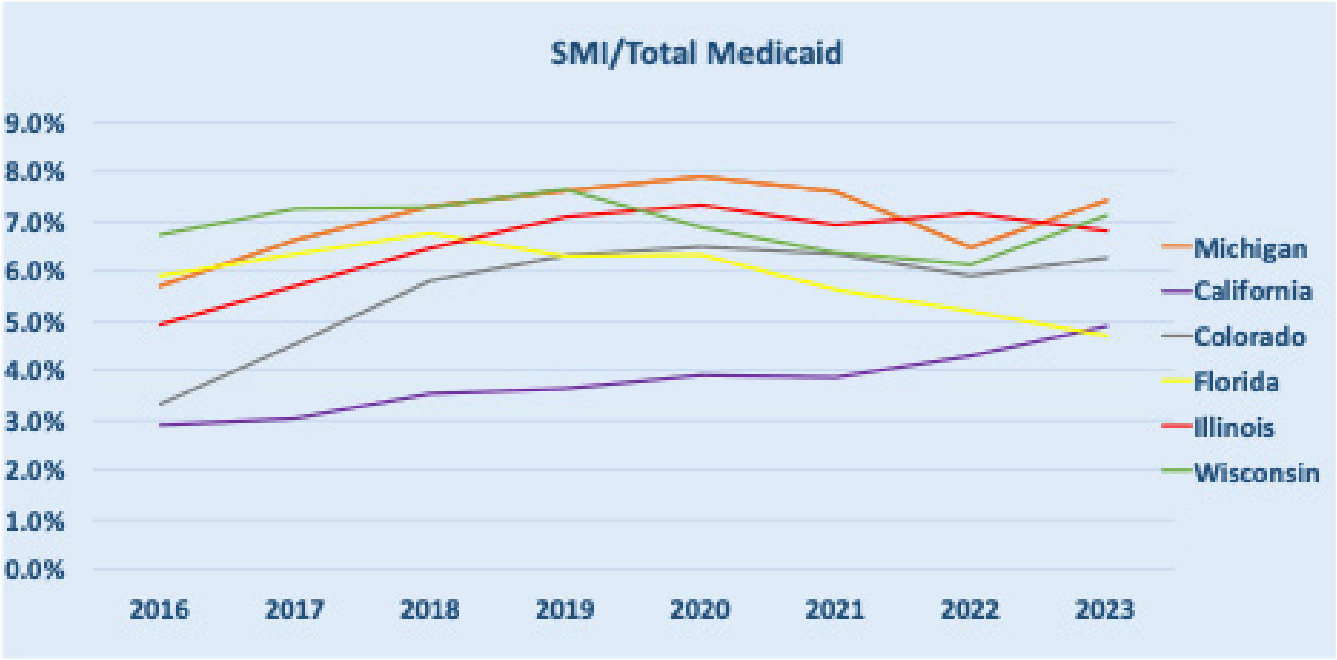

Abbreviation: SMI, serious mental illness.

Figure S3. PSM-Adjusted SMI-related Hospital Admissions Among Medicaid Beneficiaries with SMI Using APs in Michigan, California, Colorado, Florida, Illinois, and Wisconsin*

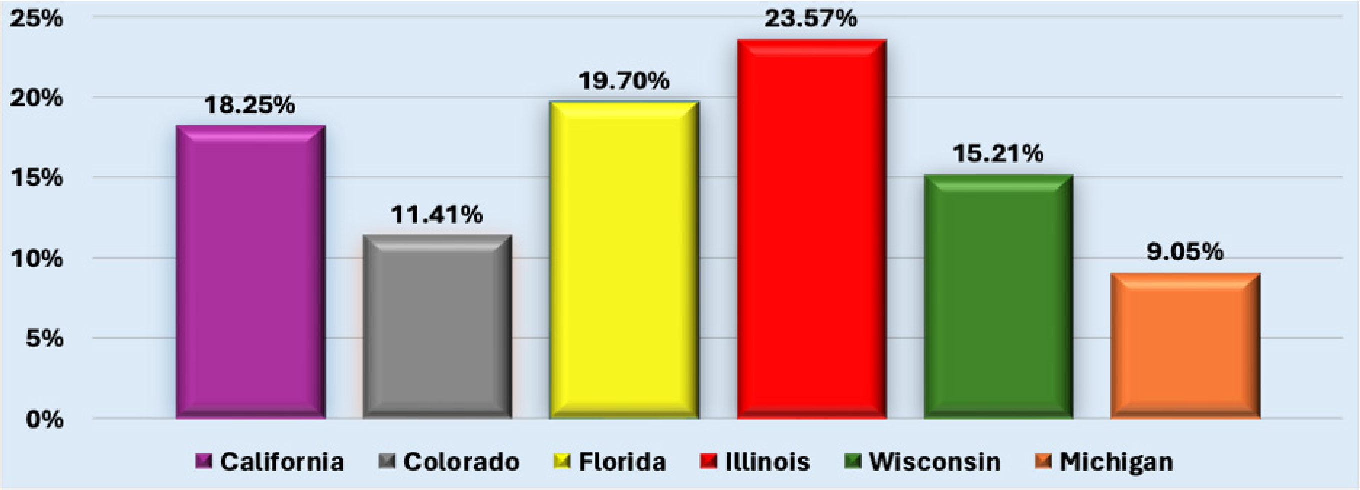

Abbreviations: AP, antipsychotic; SMI, serious mental illness.
*California, Colorado, Florida, Illinois, and Wisconsin had their own state-specific preferred drug lists, so the APs listed as preferred and non-preferred agents differed between states. Therefore, they could not be compared with each other. The value for Michigan is a weighted average of the proportion of all-cause hospital admissions among Michigan AP users compared with each state.
